# An improved competitive inhibition enzymatic immunoassay method for tetrodotoxin quantification

**DOI:** 10.1186/1480-9222-14-3

**Published:** 2012-03-12

**Authors:** Amber N Stokes, Becky L Williams, Susannah S French

**Affiliations:** 1 Department of Biology, Utah State University, 5305 Old Main Hill, Logan, Utah 84322-5305, USA; 2 Department of Biology, Utah State University Uintah Basin, 320 N 2000 W (Aggie Blvd.), Vernal, Utah 84078-4228, USA

**Keywords:** Tetrodotoxin, CIEIA, HPLC

## Abstract

Quantifying tetrodotoxin (TTX) has been a challenge in both ecological and medical research due to the cost, time and training required of most quantification techniques. Here we present a modified Competitive Inhibition Enzymatic Immunoassay for the quantification of TTX, and to aid researchers in the optimization of this technique for widespread use with a high degree of accuracy and repeatability.

## Background

Tetrodotoxin (TTX) is a low molecular weight neurotoxin that blocks the pore region of voltage-gated sodium channels [1-3] and is found in a wide array of taxa [reviewed by [4]]. The diversity of species with TTX raises questions about the ecological functions and evolutionary implications of TTX [reviewed by [5]]. Further, TTX is of concern to human health as fugu and marine gastropods are commonly consumed in Asian countries [e.g. [6,7]]. Therefore, quantification of TTX is of high importance for multiple fields of research. Traditional approaches for quantifying TTX have limited efficiency and practicality. For example, one common method of quantifying TTX is High Performance Liquid Chromatography [HPLC; reviewed by [8,9]]. HPLC is an effective means of measuring TTX but is costly, time consuming, and requires special training and expensive equipment.

A more efficient method of quantifying TTX is to use an immunoassay specific to tetrodotoxin. Several methods for Competitive Inhibition Enzymatic Immunoassays (CIEIA) or other competitive enzyme immunoassays (EIA) exist [e.g. [10-13]]. However, in our experience previously published immunoassay methods are not replicable without detailed knowledge of EIA procedures, contain errors, or report suboptimal concentrations of reagents. Here, we report a modified CIEIA procedure that employs a commercial monoclonal antibody specific to TTX for identification and quantification. Additionally, we report the repeatability between plates within a lab. This method is flexible and adaptable and could identify and quantify TTX in a range of medical or ecological studies using readily available and more affordable lab equipment and reagents.

## Results and discussion

**Figure 1:**
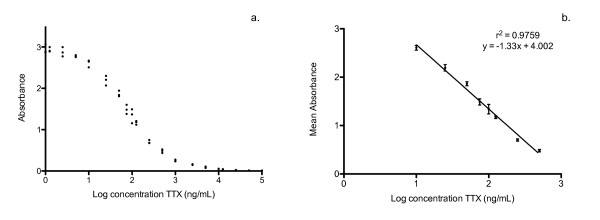
**Tetrodotoxin standard curves**. (**a**) Standard curve of tetrodotoxin using concentrations of 100,000 ng/mL through 1 ng/mL. Each sample was run in triplicate and all points are displayed to demonstrate variation between replicates. (**b**) Linear portion of standard curve (± SEM). Linear range is between 10 and 500 ng/mL.

This assay is highly repeatable, sensitive, and an accurate means of quantifying TTX (Table 1). The minimum limit of detection was 10 ng/ml (13; Figure 1a), and the linear range of the standard curve was 10-500 ng/mL with r^2 ^= 0.9759 (Figure 1b). In the linear range, the average intraplate CV for replicates was 6.38 and 7.72%, while the average interplate CV was 8.88%. The concentration of TTX at which 50% of the anti-TTX was inhibited from binding was ~75 ng/mL.

**Table 1:** Comparisons of the calculated concentrations from two different plates run the exact same way.

Concentration 1 (ng/ml)	Concentration 2 (ng/ml)	Mean (ng/ml)	Standard Deviation	CV (%)	Percent Difference
104.37	107.72	106.05	2.37	2.23	3.16

72.77	74.67	73.72	1.34	1.82	2.57

50.22	44.93	47.58	3.74	7.86	11.12

23.87	23.13	23.50	0.52	2.23	3.15

10.31	10.93	10.62	0.43	4.10	5.80

Actual standard concentrations were 100, 75, 50, 25, and 10 ng/mL from top to bottom, and were made for each plate independently

This CIEIA procedure allows for broader study of TTX-bearing organisms where a high sample volume can be screened with relatively little expense ($0.32 per sample vs. $12.00 per sample with HPLC [excluding equipment and labor]), time (7 hours vs. 48-96 hours for HPLC for 24 samples), and technical expertise. However, because TTX analogs cannot be identified with this technique, we recommend that data be augmented by HPLC or GC-MS services for a few representative samples. Testing of the primary antibodies with several TTX congeners has shown that they are specific to TTX and do not bind to these congeners [12].

Screening organisms that have yet to be tested for TTX will further our understanding of the role of TTX in ecological systems and evolution. Furthermore, this assay could facilitate rapid pathology tests in human poisoning cases that can be conducted at a wider array of research/medical facilities. Modification of existing methods was necessary to eliminate non-specific binding where possible, which is important for accurate quantification and interpretation of the results. The low variability between plates inherent to this method demonstrates that this technique is sufficiently repeatable to be widely used in a variety of medical and ecological studies.

## Conclusions

The methodology presented here modifies and refines previous methodology (Kawatsu et al. 1997; Lehman 2007; Raybould et al. 1992; Tao et al. 2010) in order to make CIEIA techniques for quantifying TTX more feasible for researchers that do not routinely perform such techniques or have access to specialized equipment (e.g., HPLC). Additionally, this CIEIA technique is more sensitive to TTX detection than HPLC from previously reported analyses [14,15]. This immunoassay has been proven useful in quantifying TTX in newts of the genus *Taricha *[16], and has quantified concentrations within the expected range of individuals quantified using HPLC previously (unpublished data). Furthermore, these methods provide the necessary methodology for eliminating issues with nonspecific binding that may occur with the technique.

## Methods

### Conjugate preparation

Conjugate preparation is significantly modified from previous work [11,12]. Specifically, TTX binds to Bovine Serum Albumin (BSA; Sigma; A7906-50 G) with formaldehyde and the BSA will tether TTX (a small hydrophilic molecule) to the plate. Because the commercial anti-TTX antibodies were created against a keyhole limpet cyanin (KLH) conjugate, the antibodies do not cross-react with BSA in the final assay [12]. Seven-hundred μL TTX (Sigma; T5651) at 1 mg/mL, 300 μL sodium acetate buffer (1 N; adjusted to pH 7.4 using 0.05 N acetic acid; Sigma; S7670), 179 μL of BSA at 33.6 mg/mL, and 41 μL of 37% formaldehyde (Fisher Scientific; AC11969) are added drop-wise to an amber glass vial (conjugate is light sensitive), in that order, and vortexed. TTX is soluble at a pH of 4-5, however previously reported methodology states that TTX should be dissolved at a pH of 7.4 in sodium acetate buffer [11,12]. We utilized1 mg TTX lyophilized in 5 mg citrate buffer and dissolved in 1 ml of ddH_2_O, which yielded the appropriate pH, with no consequences to the efficacy of the conjugate. The conjugate solution is then incubated in a shaker for three days at 37°C. Following incubation, the solution is transferred to dialysis tubing and dialyzed over a three day period at 4°C against four equally spaced 1 L-changes of phosphate buffered saline (PBS; Fisher Scientific; BP665-1). The concentration is then determined by spectrophotometry (NanoDrop ND-1000 Spectrophotometer; at 280 nm). Finished conjugate may be stored at 4°C and does not need to be lyophilized [as in [13]].

### Conjugate optimization

Optimal conjugate concentration is determined by running plates of standard curves with serial dilutions of the conjugate. Excessively concentrated conjugate results in high variation due to nonspecific binding [17]. Others [11,12] reported 2 μg/mL concentrations for anti-TTX antibodies (Hawaii Biotech) and 10 μg/mL BSA-TTXF conjugate to coat the plate. We found that using a 2 μg/mL solution of conjugate and consequently, a lower concentration of antibodies, can be used saving materials, eliminating nonspecific binding, decreasing variation, and improving the fit and accuracy of the standard curve. For each new lot of antibody purchased and used, the appropriate concentration of antibodies will have to be optimized using standard solutions of TTX and testing serially diluted anti-TTX antibodies. Both primary and secondary antibodies can be stored at 4°C or -20°C between uses.

### Extraction of TTX and preparation of standards

TTX is extracted by previously described methods [18]. Briefly, filtrates may be stored at -80°C for up to 5 yr. without degradation of TTX (CT Hanifin pers comm.). Standards are prepared using 1 mg TTX lyophilized in citrate buffer (Ascent Scientific; Asc-055) dissolved in 1 mL of a 1% solution of BSA diluted in PBS. The linear range of the curve is quite large (see results), so we use standard concentrations of 10, 50, 100, 300 and 500 ng/mL diluted in 1% BSA-PBS from the 1 mg/mL stock solution for each assay. In cases where the samples are not diluted by at least 1:2, standards are prepared by diluting in 0.1 M acetic acid rather than the 1% BSA solution. We have found that the absorbance values for acetic acid are slightly different than those of 1% BSA solution. Using acetic acid as the background for samples that are not diluted compensates for this, and does not alter the accuracy of the standard curve. All standards, samples, and stock solutions should be stored at -80°C between uses with little affect due to freeze/thaw of solutions.

### Assay set-up

**Figure 2:**
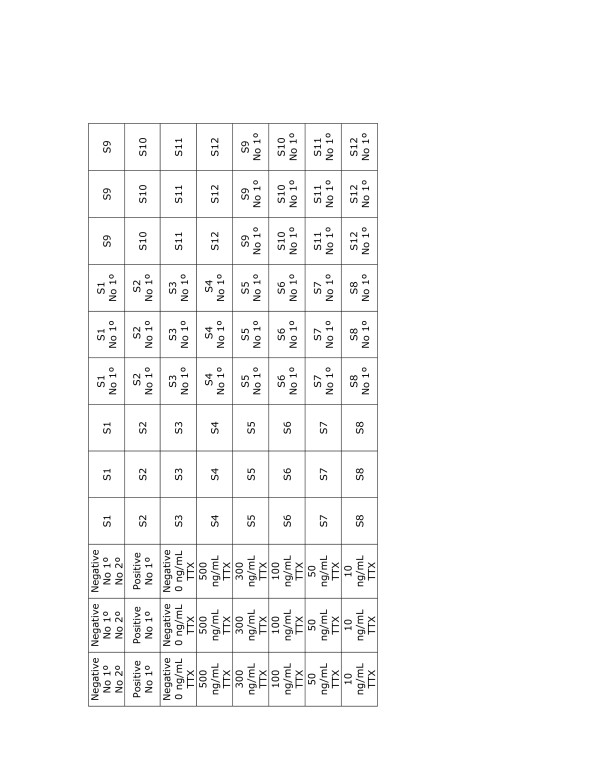
**A typical template used for plates**. Each sample starts with the letter S. Samples run without primary antibodies are used to eliminate any background noise caused from the sample itself.

Assays are run in 96-well microtiter plates (Nunc MaxiSorp, Fisher Scientific; 439454). The first of three controls is a blank and does not receive any sample, standard, or antibody (Figure 2). The second is a positive control that tests the efficacy of the alkaline-phosphatase labeled goat anti-mouse IgG+IgM (H+L) secondary antibodies (Jackson ImmunoResearch; 115-055-044). The third is a negative control, in which 1% PBS-BSA is used as a sample with no TTX. In cases where acetic acid is used to prepare standards, 0.1 M acetic acid is the negative control. This assay is very sensitive to temperature changes and should be run at approximately 25-30°C. We also report here, for the first time, that small pigment molecules not excluded during the extraction process can add to the absorbance and thus interfere with TTX quantification. Additionally, there may be non-specific secondary binding in some cases, which may give false positive results. These issues are easily circumvented by running controls of each extract with no anti-TTX antibody to measure baseline absorbance for each sample, which will be subtracted from the absorbance of the quantified sample.

### Assay procedures

The assay: (1) Each plate is coated with 100 μL conjugate diluted in PBS (2 μg/mL). The plate is incubated for one hour at room temperature (RT), and washed three times with 250 μL of PBS-T (500 μL Tween-20 (Fisher Scientific; 23336-2500) per 1 L PBS) buffer using a plate washer (Bio-Rad model 1575; may also be performed by hand). (2) We next block the plate using 200 μL of 1% PBS-BSA, incubate for one hr at RT, and again wash the plate. (3) Fifty-μL of standards or samples are added to wells in triplicate. Samples should be diluted to within the range of the standard curve (preliminary data may be collected to determine proper dilutions). (4) Fifty-μL of anti-TTX antibodies diluted to the optimal concentration (0.391 μg/ml in our case) are added to all sample and standard wells except individual extract controls, incubated one hr at RT, and washed. (5) One hundred μL of anti-mouse IgG + IgM antibodies (H + L) are added to all wells except the positive and blank controls, incubated one hr at RT, and washed. (6) Fifty μL of secondary antibodies are added to the positive control wells, and 200 μL of a 1 mg/mL pNPP solution (diluted in diethanolamine buffer: 400 mL ddH2O, 52.22 g diethanolamine (Sigma; D8885-500 G), adjusted to pH 9.80 with concentrated HCl, 0.051 g MgCl_2 _(Fisher Scientific; AC41341-0025)) is added to all of the wells. The plate is protected from light and incubated at RT for 10 minutes. (7) The plate is then read in a Bio-Rad xMark Microplate Spectrophotometer (any standard absorbance reader with the appropriate filter is sufficient) at an absorbance of 405 nm. Readings are taken every 5 minutes following the initial 10-minute reading.

### Calculations

The mean, standard deviation, and coefficient of variation (CV) for each of the standards are calculated. To back-calculate standard concentrations, mean absorbance values of the standards are plotted against the log of the known concentration for each standard. The time frame with the best standard predictions (least summed variance from known values; usually highest r^2 ^value of the regression line) is selected for sample quantification. The best time frame is usually 20-45 min. Sample values outside the range of the standard curve are diluted and re-run, re-assayed as a more concentrated extract, or reported as either below detection limit (BDL) or above detection limit (ADL) depending on whether they fall above or below the curve.. The concentrations for any samples are adjusted via dilution factor. The mean value for the negative control (BSA or acetic acid), or preferably individual extract controls, should be subtracted from the mean values of the unknowns to eliminate background noise for the most accurate final concentration.

## Competing interests

The authors declare that they have no competing interests.

## Authors' contributions

ANS ran immunoassays, fixed issues regarding repeatability and variation, and drafted and edited this manuscript. BLW ran immunoassays, gave helpful suggestions for the immunoassay, and edited this manuscript. SSF supervised this work, provided the materials and equipment necessary, gave helpful suggestions for the immunoassay, and edited this manuscript. All authors read and approve the content of the final manuscript.
